# An Integrative Small RNA–Degradome–Transcriptome Analysis Reveals Mechanisms of Heat-Induced Anther Indehiscence in Pepper

**DOI:** 10.3390/biology15020129

**Published:** 2026-01-12

**Authors:** Gang Lei, Tao Li, Kunhua Zhou, Xinjie Yuan, Yueqin Huang, Gege Li, Yu Fang, Rong Fang, Xuejun Chen

**Affiliations:** 1 Institute of Vegetables and Flowers, Jiangxi Academy of Agricultural Sciences, Nanchang 330200, China; leixxgang@163.com (G.L.); 17513272187@163.com (T.L.); zhoukunhua6080@163.com (K.Z.); yxinjie1115@163.com (X.Y.); hyq1267@163.com (Y.H.); m15907123144@163.com (G.L.); fyjxaas@163.com (Y.F.);; 2 Jiangxi Key Laboratory of Horticultural Crops (Fruit, Vegetable & Tea) Breeding, Jiangxi Academy of Agricultural Sciences, Nanchang 330200, China; 3 College of Agronomy, Jiangxi Agricultural University, Nanchang 330045, China

**Keywords:** microRNAs, transcriptome, degradome, heat stress, pepper, flower development

## Abstract

Heat threatens male fertility in crops, yet the regulatory basis of anther dehiscence under high temperatures remains unclear. We compared a heat-sensitive pepper cultivar (DL) with a heat-tolerant landrace (B021) across two anther stages using integrated transcriptome, small-RNA, degradome, co-expression, and enzymatic assays. DL showed a collapse of anther dehiscence above 34–38 °C, whereas B021 retained normal dehiscence at 39 °C, and histology revealed tapetal enlargement, premature degeneration, and locule contraction only in DL. RNA-seq indicated genotype- and stage-dependent reprogramming, with DL suppressing phenylpropanoid/cell-wall, transport, and proteostasis pathways, while B021 maintained reproductive and stress-integration programs. Small-RNA profiling and degradome sequencing identified conserved miRNA families with in vivo target cleavage, and notably, miR397 targeting a laccase gene showed stronger evidence in B021, which is consistent with controlled lignification. Functional organization of differentially expressed miRNA targets highlighted modules in respiration/redox, hormone and terpenoid metabolism, vascular–cell-wall programs, and proteostasis/osmotic buffering. WGCNA modules correlated with heat-tolerance traits converged on the same processes. Enzyme assays corroborated multi-omics predictions, with SOD, CAT, and POD activities consistently induced in B021 and limited MDA accumulation. Together, the data supports a model in which tolerant anthers sustain dehiscence under heat by coordinating secondary-wall formation, auxin/jasmonate/gibberellin crosstalk, respiratory and reactive oxygen species buffering, and protein/membrane quality control, providing tractable targets for breeding heat-resilient peppers.

## Simple Summary

High temperatures during flowering can sharply reduce pepper yield because anthers (the organs that release pollen) may not open. We compared a heat-sensitive cultivar (DL) and a heat-tolerant line (B021) under elevated temperatures. DL showed abnormal anther wall development and early tissue breakdown, producing closed anthers and poor pollen release, while B021 largely maintained normal structure and opening. To uncover the controls behind these differences, we integrated messenger-RNA profiling with microRNA (small regulatory RNA) analysis and a cleavage-based assay that confirms microRNA targets, together with gene network analysis and antioxidant measurements. B021 sustained programs that strengthen and remodel the anther wall, coordinated stress and hormone signals, and limited harmful oxidants through higher antioxidant enzyme activities (SOD, CAT, and POD) and lower membrane damage. Several conserved microRNA–target pairs were linked to these protective responses. These findings provide practical molecular markers and candidate targets to breed pepper varieties that remain fertile during heat waves.

## 1. Introduction

Global climate change has increased the frequency, duration, and intensity of heat waves, posing a profound threat to agricultural productivity and global food security [1]. High temperatures (HT) not only impair basic physiological processes, such as photosynthesis and nutrient uptake, but also severely disrupt plant reproductive development, often leading to significant yield declines [1]. The reproductive stage, which encompasses meiosis, pollen development, fertilization, and fruit set, is the most sensitive stage of the plant life cycle to high temperatures, and even brief exposure to supraspinal temperatures can cause irreversible damage [2,3].

Pepper (*Capsicum annuum* L.) is a major vegetable and spice crop valued for its nutritional and economic significance, especially in developing regions [4]. Pepper reproduction is highly sensitive to HT stress, and documented consequences include pollen sterility, anther abortion, compromised fertilization, and reduced fruit set [5]. Among these adverse effects, abnormal anther dehiscence is a key limiting factor. Anther dehiscence, the process by which mature anthers open and release viable pollen, is a precisely regulated developmental event involving complex cellular and molecular changes, including programmed cell death in the chorionic layer, degradation of the pharmacophoric septum, and coordinated remodeling of the endodermal cell wall [6]. Under HT stress, this delicate balance is disrupted, resulting in delayed or failed anther opening, which directly hinders successful pollination and fertilization [7]. Therefore, understanding the molecular mechanisms underlying the failure of anther opening in chili peppers due to high-temperature stress is not only crucial for basic biological research but also important for breeding heat-tolerant chili pepper varieties through targeted breeding strategies.

Plants have evolved complex molecular mechanisms to sense and respond to high-temperature stress. The main responses include the rapid induction of heat shock proteins (HSPs) and the production of molecular chaperones, which play key roles in maintaining protein homeostasis, refolding denatured proteins, and preventing protein aggregation under stress condition [8]. Meanwhile, high-temperature stress often leads to the overproduction of reactive oxygen species (ROS), such as superoxide anion and hydrogen peroxide, which can cause oxidative damage to cellular components [9,10]. Plants counteract this damage by activating powerful ROS scavenging systems, including enzymatic antioxidants such as superoxide dismutase (SOD), catalase (CAT), and ascorbate peroxidase (APX), as well as nonenzymatic antioxidants [11].

Hormone regulation is another key dimension of high-temperature stress response. Plant hormones, such as abscisic acid (ABA), ethylene, auxin, gibberellins and cytokinin, play complex roles in mediating stress signaling pathways and in regulating growth and development under unfavorable conditions [12]. For example, ABA often enhances stress tolerance by regulating stomatal closure and gene expression, whereas ethylene can be involved in stress perception and response and sometimes promotes senescence [13]. Growth hormone is critical for reproductive development, and alterations in its distribution or signaling under high-temperature stress can severely affect anther and pollen development [14].

In addition to transcriptional regulation, post-transcriptional regulatory mechanisms, especially those mediated by small RNAs (sRNAs), have emerged as key regulators of plant stress response. MicroRNAs (miRNAs) are a class of endogenous small non-coding RNAs that are typically 20–24 nucleotides in length, which regulate gene expression by directing RNA-induced silencing complexes (RISCs) to target mRNAs, leading to their cleavage or translational repression [15]. In the context of abiotic stresses, numerous miRNAs have been found to play key roles in regulating stress-responsive gene networks, including those involved in drought, salinity, and temperature stress [16]. These miRNAs target a variety of genes, including transcription factors, hormone signaling components, and genes involved in antioxidant defenses, thereby finely regulating adaptive responses in plants [17]. Understanding these complex transcriptional and post-transcriptional regulatory networks is critical for resolving the mechanisms underlying reproductive high-temperature tolerance in plants.

The advent of high-throughput sequencing technologies has revolutionized plant stress research, enabling comprehensive investigations of the molecular responses of plants to environmental challenges. Transcriptome analysis, often performed using RNA sequencing (RNA-seq), provides a genome-wide snapshot of gene expression profiles, enabling the identification of differentially expressed genes (DEGs) and the elucidation of transcriptional regulatory pathways under stress conditions [18]. This approach has been widely used to identify genes involved in high-temperature stress response in a variety of plant species.

Complementary to transcriptome analysis, small-RNA sequencing (sRNA-seq) identifies and quantifies miRNAs and other sRNAs, providing insights into their dynamic expression patterns and potential regulatory roles under stress [15]. By comparing sRNA profiles between tolerant and sensitive genotypes or under different stress conditions, researchers can identify key miRNAs involved in stress adaptation [19]. However, identifying the precise targets of these miRNAs is critical to understanding their functional significance.

Degradome sequencing (Degradome-seq), also known as parallel analysis of RNA ends (PARE), is a powerful technology specifically designed to validate miRNA-mediated mRNA cleavage events on a genome-wide scale [20]. By sequencing the 5′ ends of cleaved mRNA fragments, Degradome-seq can directly identify miRNA-guided cleavage sites, providing strong evidence for miRNA–target interactions [21]. Integrating transcriptome, sRNA-seq, and degradome-seq data provides a comprehensive multi-omics approach to resolving complex gene regulatory networks, enabling the identification of miRNA-mRNA modules critical for stress response. This integrated strategy provides a more comprehensive understanding of the molecular mechanisms of plant stress tolerance than any single-omics approach.

Despite significant progress in understanding plant high-temperature stress responses, the specific molecular mechanisms underlying reproductive high-temperature tolerance in Capsicum, particularly with respect to anther dehiscence, remain poorly understood. Although some studies have explored transcriptional changes in Capsicum under high-temperature stress, comprehensive and integrated multi-omics analyses targeting reproductive stages, especially anther development and dehiscence, are still lacking. miRNAs, and their target genes have not yet been fully elucidated for their precise roles in responding to high-temperature stress during the critical window of reproductive development in Capsicum [22]. Identification of key regulatory genes and pathways conferring high-temperature tolerance in pepper anthers is essential for developing effective breeding strategies.

To address these gaps, we performed an integrated multi-omics analysis that combines small-RNA (sRNA) sequencing, degradome (PARE) profiling, and transcriptome (RNA-seq) profiling of anthers from heat-tolerant and heat-sensitive Capsicum annuum genotypes exposed to high-temperature stress during the reproductive stage. This design enabled systematic discovery of differentially expressed miRNAs and mRNAs, experimental validation of miRNA–target cleavage events, and reconstruction of an anther-specific miRNA–mRNA regulatory network associated with heat responses. Leveraging network topology together with expression and degradome evidence, we resolved putative hub genes and regulatory modules implicated in endothelial secondary-wall thickening, stomium remodeling, and the execution of anther dehiscence under heat. Collectively, the study delineates the multilayered regulatory architecture of reproductive heat tolerance in pepper and yields tractable molecular candidates to accelerate breeding of climate-resilient cultivars.

## 2. Materials and Methods

### 2.1. Plant Materials, Cultivation, and Heat Stress Treatment

Two pepper accessions with differential heat tolerance at the reproductive phase were used in this study. The heat-sensitive genotype ‘DL’ from southwest China is extremely sensitive to heat stress at the reproductive phase under a high-temperature (HT) environment. The heat-tolerant genotype ‘B021’, a landrace originally collected from southeast China, is highly tolerant to heat stress and can bloom and fruit under HT conditions at the reproductive phase. Plants at the four-to-six true leaf stage were transplanted into buckets (25 cm height, with an interior diameter of 30 cm) with equal paddy soil mixed with growth substrate (Pindstrup, Ryomgaard, Denmark) in equal volumes. One strong seedling was planted per bucket, with 12 buckets per genotype, and kept at 28.0/25.0 °C (14 h day/10 h night) and 60% humidity (Figure S1). At the full flowering stage, all visible floral buds on the plants were removed. And then plants for HT treatment were moved into chambers and maintained at a temperature of 38.0  ±  0.5 °C (treatment) or 28.0  ±  0.5 °C (control) for the light period (14 h) and 28.0  ±  0.5 °C (both treatment and control) for the dark period (10 h). Three biological replicates of the temperature treatments were grown under the same conditions. After 48 h of treatment, the floral buds with different sizes represented different developmental stages (pollen mother cell stage and tetrad stage) (Figure S1) and were harvested, packed in aluminum foil, and flash-frozen in liquid nitrogen until further use. A total of 24 pepper floral bud samples were harvested, i.e., treatments (B021/DL_T1_1, B021/DL_T1_2, and B021/DL_T1_3) and controls (B021/DL_CK1_1, B021/DL_CK1_2, and B021/DL_CK1_3) of the three replicates of the heat-tolerant (B021)/heat-sensitive (DL) genotype at the pollen mother cell stage, and treatments (B021/DL_T2_1, B021/DL_T2_2, and B021/DL_T2_3) and controls (B021/DL_CK2_1, B021/DL_CK2_2, and B021/DL_CK2_3) of the three replicates of B021/DL at the tetrad stage.

To determine the effect of HT stress on the heat-tolerant and -sensitive genotypes, plants with different temperature treatments (34.5 °C, 35.5 °C, 36.5 °C, 37.5 °C, 38.0 °C, and 39.0 °C/48 h) were moved to normal growth conditions until flowering. Then, anther dehiscence rate (ADR) and pollen viability were measured. ADR was calculated using the formula ADR (%) = 100% × Number of dehiscent anthers/Number of anthers investigated. Pollen viability was measured with the 1,2,3-triphenyl tetrazolium choride (TTC) method.

### 2.2. Total RNA Extraction

Total RNA was isolated and purified using TRIzol reagent (Invitrogen, Carlsbad, CA, USA), following the manufacturer’s procedure. The RNA amount and purity of each sample were quantified using NanoDrop ND-1000 (NanoDrop, Wilmington, DE, USA). The RNA integrity was assessed by the Bioanalyzer 2100 system (Agilent Technologies, Palo Alto, CA, USA) with RIN number > 7.0. The total RNA extracted from each sample was utilized to construct the library for transcriptome, small-RNA, and degradome sequencing.

### 2.3. Library Construction, Transcriptome Sequencing, and Transcript Assembly

Total RNA was purified with oligo (dT) magnetic beads to obtain mRNA. After purifying, the mRNA was fragmented into small pieces via the Magnesium RNA Fragmentation Module (NEB, cat. E6150S, Ipswich, MA, USA). Then, the cleaved mRNA fragments were used to form the final cDNA library according to the previous methods [23]. The average insert size for the final cDNA library was 300 ± 50 bp. Finally, the pair-end sequencing was performed on an Illumina Novaseq™ 6000 platform (Illumina, San Diego, CA, USA) to generate paired-end reads by the I Gene Book (Wuhan, China) according to the manufacturer’s instructions.

First, raw data were preprocessed by removing reads that contained adaptor contamination, low-quality bases, and undetermined bases using Cutadapt 5.1 (parameter setting: -a R1_adpter -A R2_adpter -m 20 –max-n 0.05 -q 20) [24], and the sequence quality was verified using FastQC (v0.12.1). Then, the retained clean reads were mapped to the CA59 reference genome [25] using HISAT2 software (v2.0.1-beta) [26], and the mapped reads were assembled using StringTie (v2.2.0) [27].

### 2.4. Expression Analysis and Annotation

Expression levels of all transcripts were estimated using featureCounts [28] and normalized as reads per kilobase of transcript per million mapped reads (RPKM). The differentially expressed genes (DEGs) were identified with |log2 (fold change)| ≥ 1 and false discovery rate (FDR) < 0.05 by the R package edgeR (v4.8.2) [29]. Then, the DEGs were annotated based on the NCBI nonredundant (NR), Gene Ontology (GO), and Kyoto Encyclopedia of Genes and Genomes (KEGG) databases, using BLASTX (v2.17.0) algorithms with a significant threshold E-value < 1 × 10^−5^. Finally, GO enrichment and KEGG enrichment analysis of DEGs were performed using in-house Perl scripts.

### 2.5. Small-RNA Sequencing and miRNA Identification

Approximately 1 ug of total RNA was used to prepare a small RNA library according to the protocol of TruSeq Small RNA Sample Prep Kits (Illumina, San Diego, CA, USA). And then we performed the single-end sequencing (1 × 50 bp) on an Illumina Hiseq2500 at the LC-BIO (Hangzhou, China), following the vendor’s recommended protocol. The raw data were processed using an in-house program, ACGT101-miR (LC Sciences, Houston, TX, USA), to remove adapter dimers, junk, low complexities, common RNA families (rRNA, tRNA, snRNA, and snoRNA) (http://rfam.sanger.ac.uk/ (accessed on 15 January 2025)), repeats (http://www.girinst.org/repbase (accessed on 15 January 2025)), and sequences < 18 nt or >25 nt in length. Subsequently, the unique sequences with a length of 18~25 nt were mapped to miRNA sequences in miRBase 22.0 (http://www.mirbase.org/ (accessed on 15 January 2025)). Mapping was also performed on pre-miRNA against pepper genomic data. The unique sequences that aligned to the known miRNA sequences in miRbase 22.0 were identified as known miRNAs. Secondary structure of pre-miRNAs was presented as a hairpin, including 5p- and 3p-derived miRNA. The unique sequences mapping to the other arm of the pre-miRNA sequences, which were not annotated in the miRbase 22.0, were considered to be 5p- or 3p-derived miRNA candidates. The remaining unmapped sequences were matched to the pepper genomic sequences in search of candidate novel miRNAs. To identify the results of putative miRNAs in pepper, all the obtained miRNAs were used to predict the secondary structures using RNAfold software (http://rna.tbi.univie.ac.at/cgi-bin/RNAWebSuite/RNAfold.cgi (accessed on 15 January 2025)). The non-coding sequences that could form a stem-loop structure and meet the standard of miRNA prediction were regarded as the true miRNAs of pepper. The differential expression of miRNAs based on normalized deep-sequencing counts was analyzed by selectively using Student’s *t*-test. The significance threshold was set at 0.05. Then, differentially expressed miRNAs were selected with |log2 (fold change)| ≥ 1 and *p* value < 0.05.

### 2.6. Degradome Sequencing and Target Identification

The RNA samples of B021 and DL collected at different stages under control/HT treatment conditions were, respectively, mixed together to generate two degradome libraries. The degradome libraries were constructed according to the method described previously [30]. Then, the constructed library was sequenced (1 × 50 bp single-end sequencing) on an Illumina HiSeq 2500 system (LC-Bio, Hangzhou, China), following the vendor’s procedure.

After degradome sequencing, adapter and low-quality sequences were removed from raw data using ACGT101-DEG (LC Sciences, Houston, TX, USA). The remaining 20 or 21 nt high-quality sequences were aligned with the sequences of cDNA in the database of pepper to produce the degradome density file. Then, the mRNA-sRNA degradation sites were predicted using CleaveLand (v4.0) [31], and oligomap was used to accurately match the mRNAs from different species to the pepper degradome sequence [32]. The sequences that matched the target genes in the miRNA library were collected and scored according to the plant miRNA/target pairing standard using the Needle program [33]. Based on the signature abundance at each occupied transcription position, the identified transcripts were divided into five categories (0, 1, 2, 3, and 4). Finally, the function of the most abundant miRNA target was analyzed through GO and KEGG pathway analyses.

### 2.7. Integrated Analysis of Transcriptome, miRNA, and Degradome Data

Integration was performed within the four predefined contrasts (B021_T1 heat vs. control, B021_T2 heat vs. control, DL_T1 heat vs. control, and DL_T2 heat vs. control). For each contrast, we first compiled the list of differentially expressed miRNAs (FDR 0.05) and differentially expressed genes (absolute log2 fold change at least 1 and FDR 0.05). Degradome sequencing was used to identify miRNA-guided cleavage events and assign confidence categories 0–4 unless stated otherwise, and categories less than or equal to 2 with a *p* value of less than 0.05 were considered strong evidence. High-confidence miRNA–mRNA pairs were defined as the intersection of differentially expressed miRNAs, degradome-validated targets, and differentially expressed genes in the same contrast. When multiple cleavage sites or transcript isoforms were available for a given pair, we retained the event with the strongest degradome support based on category, *p* value, and peak height, and reported all events in Supplementary Data. Directionality was assessed by verifying inverse changes between the miRNA and its target. The validated pairs were assembled into a bipartite miRNA–mRNA network, and communities (modules) were identified by standard community detection (Louvain or Leiden) and annotated by Gene Ontology and KEGG enrichment using all expressed genes as background and FDR 0.05 as the significance threshold. Hub regulators within trait-relevant modules were nominated by high centrality measures. Robustness was checked by repeating the integration using only categories with less than or equal to 2 edges and by varying the differential expression threshold within a narrow range. Software and parameters used in this integrative analysis were summarized to improve reproducibility. Raw read quality was checked using FastQC (v0.10.1). The small RNA analysis pipeline was conducted using ACGT101-miR (v4.2) and ACGTUNAfold (v3.7), and miRNA target/cleavage-site prediction was performed using CleaveLand (v4.0). Degradome analysis was carried out using ACGT101-DGD-v4.0 (v4.1). Functional annotation/enrichment and network visualization were performed using R (v3.6.0) and Cytoscape (v3.10.3).

### 2.8. Weighted Gene Co-Expression Network Analysis (WGCNA)

Normalized gene expression matrices from all anther samples (log2-transformed expression values; low-abundance genes removed) were analyzed in R using the WGCNA package (v1.69-81) [33]. First, hierarchical clustering of samples was performed to detect potential outliers. Second, a signed co-expression network was constructed using Pearson correlation. The soft-thresholding power (β) was selected using the scale-free topology criterion (target R^2^ ≥ 0.8 while maintaining sufficient mean connectivity). Third, the adjacency matrix was transformed into a topological overlap matrix (TOM), and genes were hierarchically clustered based on 1–TOM dissimilarity. Fourth, initial gene modules were detected using the dynamic tree cut algorithm (minimum module size > 30). Closely related modules were then merged when the correlation between their module eigengenes was high (eigengene correlation > 0.75, equivalent to merge cut height < 0.25). For each module, the module eigengene (ME) was calculated and correlated with experimental traits (genotype, treatment, stage, ADR, and vigor pollen rate (VPR)) using Pearson correlation; two-sided *p* values were corrected by the Benjamini–Hochberg method. To prioritize key regulators, hub genes were defined as genes with high module membership.

### 2.9. Real-Time Quantitative PCR Analysis

Total RNA from anther tissues was extracted with TransZol Up Plus (TransGen, ER501-01, Beijing, China) and quantified on a Nanophotometer; integrity was checked by agarose electrophoresis. Three biological replicates per condition were processed. For mRNA assays, first-strand cDNA was synthesized from 0.5 to 1 µg total RNA using TransScript^®^ Uni All-in-One First-Strand cDNA Synthesis SuperMix for qPCR (One-Step gDNA Removal) (TransGen, AU341, Beijing, China), according to the manufacturer’s instructions. Quantitative PCR was performed on a CFX Connect real-time system (Bio-Rad, Hercules, CA, USA) using MagicSYBR Mixture (Cwbiotech, CW3008, Taizhou, China). Each 20 µL reaction contained 10 µL 2× MagicSYBR, 0.4 µL each of forward and reverse primers (10 µM), 1 µL cDNA, and nuclease-free water to volume. Cycling conditions (three-step protocol) were 95 °C for 10 min, 40 cycles of 95 °C for 10 s, 60 °C for 30 s, and 72 °C for 30 s, and a melt-curve analysis was performed to verify amplicon specificity.

For miRNA quantification, cDNA was generated with the same SuperMix using miRNA-specific primers (forward, miRNA-specific, reverse, and universal), and qPCR was run with MagicSYBR as above. Primer pairs are provided in Supplemental Table S6, and representative targets included CaDEM01G00700, CaDEM02G11160, CaDEM03G45400, and CaDEM04G00350. UBQ5 and β-TUB served as internal reference genes for mRNA assays; miRNA assays used the corresponding small-RNA primer set with a universal reverse primer. Fluorescence thresholds were set automatically. Relative expression was calculated by the 2^−ΔΔCt^ method after confirming single-peak melt curves.

### 2.10. Determination of Antioxidant Enzyme Activities and Lipid Peroxidation

Fresh anthers (~0.10 g per replicate) were homogenized on ice in the extraction buffer supplied with each commercial kit (*w*/*v* ≈ 1:10) and clarified at 12,000 rpm for 10 min at 4 °C, and the supernatants were used immediately. Three biological replicates were analyzed per condition with in-plate blanks and technical duplicates, and protein content was determined by BCA for activity normalization. Catalase (CAT) was assayed with kit ADS-W-KY002-48 (Addison Biotech, Yancheng, China) by quantifying residual H_2_O_2_ with a chromogenic probe at 510 nm and calculating activity as μmol min^−1^ mg^−1^ protein according to the manufacturer’s equation. Superoxide dismutase (SOD) activity was measured using the WST-8 method (Addison Biotech, ADS-W-KY011, Yancheng, China): superoxide generated by the xanthine/xanthine-oxidase system reduces WST-8 to a formazan detected at 450 nm, inhibition by SOD was used to compute units (U mg^−1^ protein), where 50% inhibition defines one unit in the reaction system. Peroxidase (POD) was quantified with kit ADS-W-KY003-48 (Addison Biotech, Yancheng, China) via guaiacol oxidation in the presence of H_2_O_2_, monitoring the rate of increase at 470 nm (reported as ΔOD_470_ min^−1^ mg^−1^ protein). Malondialdehyde (MDA), as an index of lipid peroxidation, was determined using the thiobarbituric-acid (TBA) method (90–95 °C, 30 min), and concentrations were calculated from ΔA = A_532_ − A_600_ with the vendor’s extinction coefficient and expressed as nmol g^−1^ fresh weight (Addison Biotech, ADS-W-YH002, Yancheng, China).

## 3. Results

### 3.1. Contrasting Anther Dehiscence Responses to Heat Stress in Pepper

**Figure 1:**
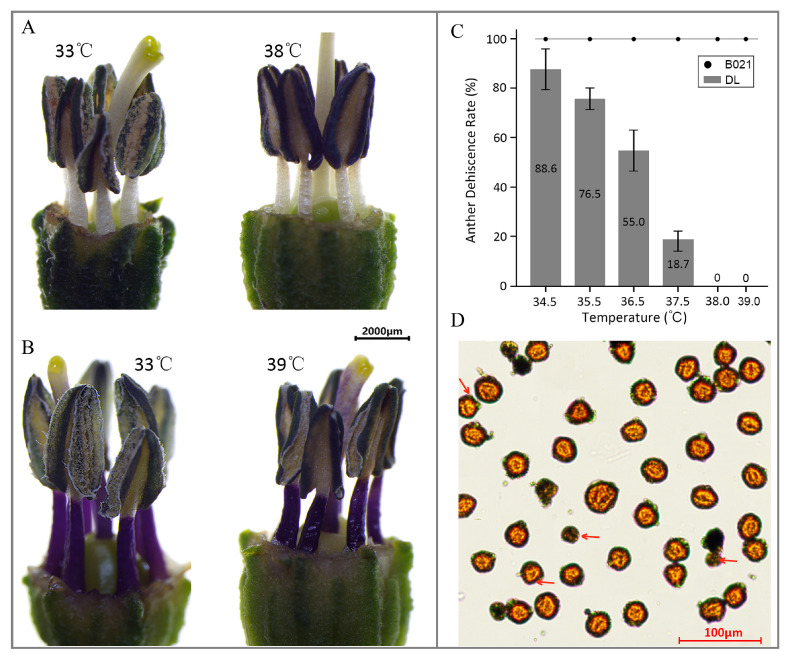
**Anther dehiscence: Differential responses of two pepper cultivars to high temperature.** (**A**) Phenotype of the heat-sensitive cultivar ‘DL’ under high-temperature (HT) stress. (**B**) Phenotype of the heat-tolerant cultivar ‘B021’ under HT stress. (**C**) Anther dehiscence rate (ADR) under different temperature conditions. (**D**) Pollen viability phenotype of the heat-tolerant cultivar at 39 °C.

The heat-sensitive cultivar ‘DL’ (southwest China) exhibited an evident impairment in anther dehiscence (Figure 1A). By contrast, the heat-tolerant landrace ‘B021’ (southeast China) maintained normal flowering and fruit set under HT (Figure 1B). Consistently, the anther dehiscence rate (ADR) displayed a temperature-dependent change, with clear divergence between the two cultivars across the tested temperature gradient (Figure 1C). Moreover, at 39 °C, the heat-tolerant cultivar maintained pollen viability, although shrunken pollen grains could still be observed and are highlighted by arrows in the microscopy image (Figure 1D).

To further determine the effect of HT on anther development, anatomical observations were conducted on anthers at different development stages after HT treatment. As shown in Figure S1, during the pollen mother cell (PMC) stage, obvious enlargement of tapetum cells squeezes the pollen mother cell, with the result that no normal pollen mother cells were formed in the heat-sensitive genotype. At the tetrad stage, the tapetum and pollen mother cells were significantly degraded, and the anther chamber also showed obvious contraction. At the mature pollen grain stage, further contraction of the anther chamber causes the cell degradation residue to squeeze into a dark band, which is consistent with the observed shriveled appearance of the anthers. Meanwhile, normal development was observed at all stages in the anthers of heat-tolerant genotype B021 (Figure S1).

### 3.2. Heat Stress Triggers Genotype- and Stage-Dependent Transcriptome Reprogramming in Pepper Anthers

We profiled 24 RNA-seq libraries from two genotypes (heat-tolerant B021 and heat-sensitive DL), two anther stages (T1, pollen-mother-cell, and T2, tetrad), and two temperature regimes. Sequencing generated 1120.21 million raw reads: 99.31% survived QC. On average, 44.25 million reads per library (95.48% mapped) quantified 39,207 expressed genes, with biological replicates tightly correlated (Pearson’s r ≥ 0.97) (Figure S2), indicating high-quality sequencing data (Supplemental Table S1). Across four contrasts (B021_T1 vs. CK1, B021_T2 vs. CK2, DL_T1 vs. CK1, and DL_T2 vs. CK2), we detected 5745 DEGs at |log2FC| > 1 and FDR ≤ 0.05 (Figure S3, Supplemental Table S2). At T1, DL exhibited an overall down-regulation trend of gene expression under high-temperature stress (2050 DEGs: 728 up, 1322 down) than B021 (973 DEGs: 544 up, 429 down), which is consistent with the sensitivity of DL anthers to heat. At T2, both genotypes showed expanded responses—B021 (2817 DEGs: 1865 up, 952 down) and DL (3245 DEGs: 2006 up, 1239 down).

**Figure 2:**
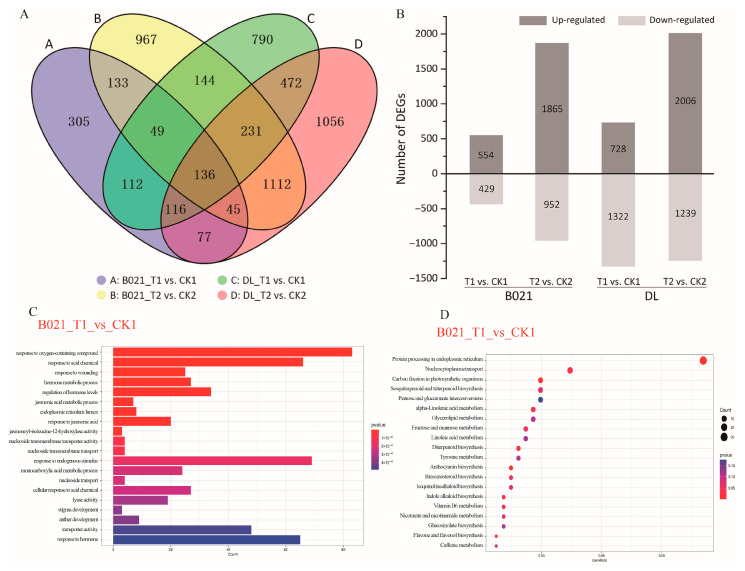
**Differential gene expression and functional enrichment analysis under high-temperature stress.** (**A**) Numbers of differentially expressed genes (DEGs) identified in four predefined contrasts (B021_T1 vs. CK1, B021_T2 vs. CK2, DL_T1 vs. CK1, and DL_T2 vs. CK2). (**B**) Partition of DEGs into up- and down-regulated sets for each contrast, highlighting the stronger early suppression in DL at the pollen-mother-cell (T1) stage. (**C**,**D**) GO and KEGG enrichment plots. Abbreviations: T1, pollen-mother-cell stage; T2, tetrad stage; CK, control; and HT, heat treatment.

Overlap analysis revealed a core set of 136 DEGs shared by all four comparisons, alongside large stage-specific gene sets, particularly at T2 (e.g., B021_T2 unique ≈ 967, DL_T2 unique ≈ 1056). Bar charts further highlight the disproportionate down-regulation in DL at T1 and the broad induction at T2 in both genotypes (Figure 2A,B). Functional profiling uncovered divergent molecular strategies. At T1 in B021, up-regulated genes were enriched for floral organ development (anther/androecium/stamen) and stress-response terms (e.g., temperature, wounding, and pathogen), together with KEGG categories in hormone signaling, carbon fixation/energy metabolism, and plant–pathogen interaction. Selected secondary-metabolism routes (e.g., carotenoid, brassinosteroid biosynthesis) were down-weighted. In contrast, DL showed down-regulation of transmembrane transport, pollen wall assembly, and phenylpropanoid/secondary-metabolism programs, alongside altered carbohydrate remobilization and MAPK signaling, indicating early compromise of wall construction and transport capacity (Figure 2C,D and Figure S4). At T2, both genotypes up-regulated modules linked to RNA/nucleic-acid metabolism and associated catalytic activities, reflecting intensified post-transcriptional and energetic demands. The key divergence lay in down-regulated pathways: B021 preferentially deemphasized “other secondary metabolism,” which is consistent with resource reallocation, whereas DL suppressed cell-wall biogenesis, responses to oxygen-containing compounds/temperature/chitin, and elements of proteostasis and lipid metabolism, aligning with continued locule contraction and failed anther opening under heat (Figure 2C,D and Figure S4).

Notably, several GO terms were commonly enriched in both the heat-tolerant cultivar B021 and the heat-sensitive cultivar DL, including endonuclease activity, RNA modification, and RNA binding. These shared functional categories are closely associated with RNA metabolism and post-transcriptional regulation, indicating that general molecular mechanisms related to pollen development and physiological maintenance may be broadly affected by high-temperature stress in pepper. Collectively, the data defines a genotype- and stage-dependent remodeling of the anther transcriptome: B021 maintains reproductive-developmental programs and stress-integration networks from PMC onward, whereas DL undergoes multi-axis suppression involving phenylpropanoid/cell-wall assembly, transport, and protein homeostasis. These transcriptomic patterns provide a mechanistic basis for the contrasting anther dehiscence phenotypes under heat stress.

### 3.3. Heat-Induced Remodeling of the Anther miRNA Layer

**Figure 3:**
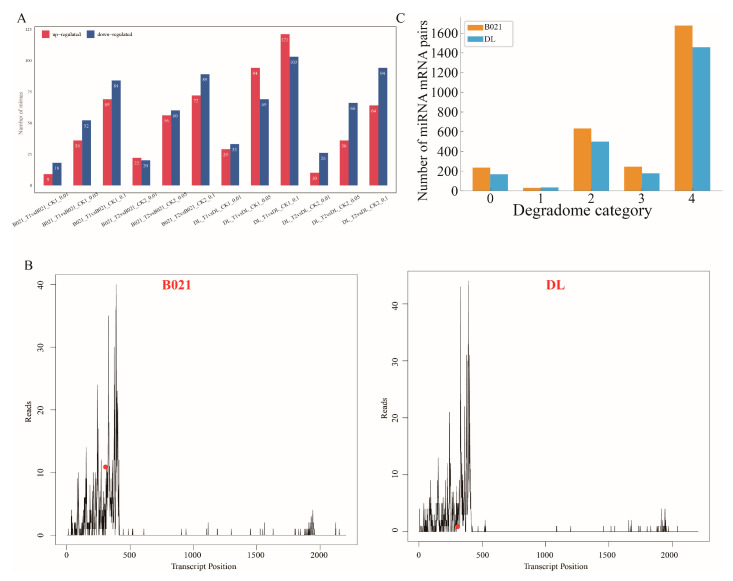
**Heat-responsive miRNA remodeling and degradome-supported targeting in pepper anthers.** (**A**) Numbers of differentially expressed miRNAs (DEmiRs) detected under three significance thresholds (FDR  <  0.01/0.05/0.10) for the four predefined contrasts (B021_T1 vs. CK1, B021_T2 vs. CK2, DL_T1 vs. CK1, and DL_T2 vs. CK2), partitioned into up- and down-regulated sets. (**B**) Representative target plots (T-plots) showing the 5′ ends of degradome tags aligned along the target transcript in B021 and DL. The peak at the predicted cleavage position indicates in vivo miRNA-guided slicing. (**C**) Summary of degradome-validated miRNA–mRNA interactions: total counts of DEmiR–DEG pairs recovered in B021 and DL, with the distribution of confidence categories (0–4) assigned by degradome analysis.

Across four contrasts (B021_T1 vs. CK1, B021_T2 vs. CK2, DL_T1 vs. CK1, and DL_T2 vs. CK2), heat stress reprogrammed the anther miRNA landscape in a genotype- and stage-dependent manner (Figure 3A and Figure S5). We observed a larger early (PMC) perturbation in the heat-sensitive genotype DL and broader responses at the tetrad stage in both genotypes (Figure 3A). Family-level profiles revealed that conserved miRNA families with direct relevance to anther dehiscence—miR397/408/858 (laccases and secondary-wall modules), miR167/160/390 (auxin signaling and TAS3), miR319/159 (TCP/MYB, jasmonate crosstalk), miR164 (NAC, wall/PCD), miR172 (AP2), and miR398 (ROS homeostasis)—displayed distinct patterns between B021 and DL (Figure S5, Supplemental Table S3). Degradome profiling validated numerous miRNA–mRNA pairs spanning categories 0–4, thereby providing in vivo evidence for miRNA-guided cleavage under heat (Figure 3C, Supplemental Tables S4 and S5). A representative T-plot confirmed miR397-guided cleavage of a laccase (*LAC*) transcript under heat stress (Figure 3B).

Taken together, these results indicate that heat stress does not trigger a generic miRNA response; instead, it reconfigures specific miRNA–target axes that are mechanistically tied to anther dehiscence. In B021, modules, such as miR397-*LAC* (secondary-wall lignification), miR167/160-*ARF*/*TAS3* (auxin), and miR319/159-*TCP*/*MYB* (jasmonate crosstalk), together with ROS-linked families (e.g., miR398), maintain endothecial secondary-wall thickening, support timely stomium/septurn remodeling, and preserve hormone–redox balance under heat. By contrast, DL exhibits weaker or mistimed regulation along these axes, consistent with failed dehiscence and reduced ADR. These miRNA–target modules, therefore, provide mechanistic entry points and tractable candidates for breeding heat-resilient pepper cultivars.

### 3.4. Heat-Responsive miRNA–mRNA Modules Underlying Anther Dehiscence

**Figure 4:**
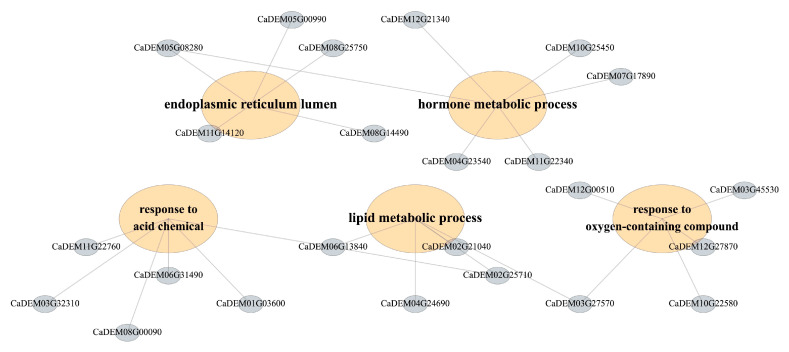
**Functional organization of DEmiR targets into process-level modules under heat.** Detailed genes for enrichment analysis.

To explore the potential biological functions associated with heat-responsive miRNA-mediated regulation, we conducted GO and KEGG enrichment analyses of the predicted target genes of differentially expressed miRNAs across the four contrasts. Across the four contrasts, enrichment analysis of the targets of differentially expressed miRNAs identified coherent process-level modules (Figure 4). For KEGG enrichment, to avoid unstable estimates driven by very small gene sets, we report and interpret pathways supported by at least three target genes (gene count ≥ 3) in the main Figure/text, while lower-count pathways are provided in the Supplementary Tables for completeness. Terms associated with mitochondrial energy metabolism—including aerobic respiration, tricarboxylic-acid cycle, citrate/dicarboxylic-acid metabolism, and cellular respiration—were significantly over-represented, together with nitrogen assimilation/amino-acid metabolism and nuclear transport/ribosome biogenesis (nuclear import, ribosomal-subunit export). Developmental categories related to phloem/xylem histogenesis and meristem initiation were also enriched (Figure S6). A second module comprised hormone and terpenoid metabolism/signaling, encompassing diterpenoid/terpenoid and gibberellin biosynthesis, oxidoreductase activity acting on NAD(P)H, and responses to oxidative stress and light. A third module highlighted lipid catabolism and membrane remodeling (mono/di/triacylglycerol catabolic processes) together with osmotic/salt-stress responses and a proteostasis/ER–peroxisome quality-control axis. Taken together, the enrichment profile in Figure 4 resolves the regulatory landscape of DEmiR targets into energy/redox, hormone/terpenoid, vascular–cell-wall, and membrane/proteostasis–osmotic modules.

### 3.5. WGCNA Co-Expression Network Analysis

**Figure 5:**
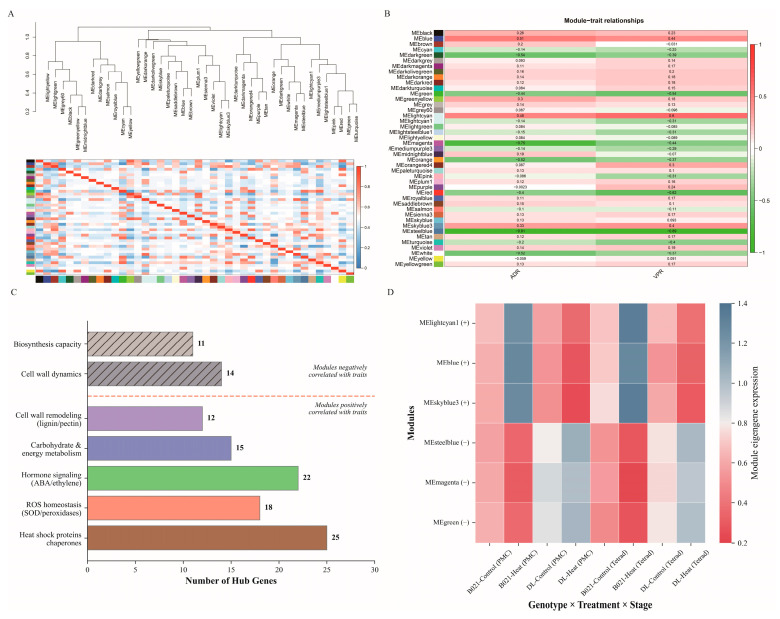
**WGCNA modules associated with heat tolerance and pollen performance.** (**A**) Sample and module clustering results based on hierarchical clustering of the topological overlap matrix, showing module assignments (color bands) and the segregation of co-expressed gene sets. (**B**) Module–trait relationships, presenting Pearson correlations (and corresponding values) between module eigengenes and phenotypic traits (ADR, vigor pollen rate), with positively and negatively associated modules indicated by the color scale. (**C**) Prioritization of hub genes within trait-associated modules based on high module membership and intra-module connectivity. (**D**) Eigengene expression profiles across genotypes, developmental stages, and treatment conditions, illustrating genotype- and stage-specific deployment of protective programs under heat.

Weighted gene co-expression network analysis divided the transcriptome into discrete modules with consistent expression patterns and clear associations with traits. A hierarchical clustering map of modules based on clustering of trait genes (Figure 5A) revealed a well-structured hierarchy of modules, which included modules of the classical color categories of blue, brown, lime green, green, magenta, steel blue, azure3, and light cyan1, suggesting that the set of co-expressed genes has a robust topological segregation feature. Trait association analysis identified a set of modules strongly associated with the reproductive heat response (Figure 5B). These opposing trends suggest that the positively correlated modules capture protective programs active in heat-tolerant fertile anthers, whereas the negatively correlated modules represent biological processes that are attenuated or maladapted under heat stress.

Based on the intra-module connectivity (kWithin) and module membership (kME) metrics, key hub genes were identified in the trait-related modules (Figure 5C). For example, the most trait-positive module MElightcyan contained hub candidates with clear relevance to reproductive heat resilience, including miR156–*SPL*-associated transcriptional regulators (known to be required for male fertility and proper anther development) and proteostasis-related chaperone components (e.g., HSP70/90) that are commonly mobilized to protect pollen/anther function under heat stress. In contrast, the strongly trait-negative module MEsteelblue was enriched for hub candidates related to cell-wall/secondary-wall dynamics, including a miR397–*laccase*-associated lignification node and pathways linked to endothecium thickening and anther dehiscence (e.g., MYB26-related regulation) (Figure S6). These representative hub genes help illustrate that the positive modules capture protective programs supporting normal dehiscence under heat, whereas the negative modules reflect processes that are suppressed or maladaptive in heat-sensitive anthers. In the positively correlated modules (MElightcyan, MEblue, and MEskyblue3), the top hub genes were enriched for functions related to heat tolerance and anther function, including protein quality control (heat shock proteins/chaperone auxin), reactive oxygen species homeostasis (superoxide dismutase/peroxidase/ascorbate peroxidase pathway), hormone signaling components (abscisic acid/ethylene/growth factor nodes), carbohydrate and energy metabolism, and cell wall-modifying enzymes associated with anther dehiscence (phenylpropane/lignin and pectin remodeling factors). In contrast, hub genes in the negatively correlated modules (MEsteelblue, MEmagenta, and MEgreen) were enriched for biological processes that were downregulated under heat stress, including categories indicative of impaired cell-wall dynamics and diminished biosynthesis capacity, consistent with impaired anther dehiscence under sensitive conditions. Together, these hub gene sets outline complementary regulatory hierarchies that may collectively determine the success or failure of anther dehiscence under high temperature.

Module trait gene expression profiles further confirmed genotype- and development-mental stage-specific behaviors consistent with correlation mapping (Figure 5D). Modules positively correlated with traits showed elevated levels of characterized gene expression in heat-treated samples from heat-tolerant genotypes (B021) and reduced levels in heat-treated samples from sensitive genotypes (DL), while negatively correlated modules showed the opposite pattern, with higher levels of characterized genes in DL control samples that were significantly suppressed under heat treatment. In addition, several modules exhibited distinct stage-specific peaks, suggesting that deployment of the protective network is time-specific at the pollen mother cell and tetrad stage. These consistent trends in characterized genes, combined with trait correlation analyses and hub-gene composition, support the model that in heat-tolerant plants, activation of protein homeostasis, redox buffering, hormone signaling, energy/carbon partitioning, and cell wall remodeling modules underlie normal anther dehiscence, whereas inhibition of these modules leads to failure of anther dehiscence in sensitive plants.

### 3.6. Key Gene Expression Validation by RT-qPCR

To experimentally verify the reliability of the multi-omics findings, we selected eight key genes for quantitative real-time fluorescence quantitative PCR (RT-qPCR) analysis. These genes were mainly associated with miR156 and miR166 regulatory modules and were highly differentially expressed or central in the co-expression network. The genes included those directly regulated by miRNA targets and involved in anther development and heat stress response, as listed in Supplemental Table S6.

**Figure 6:**
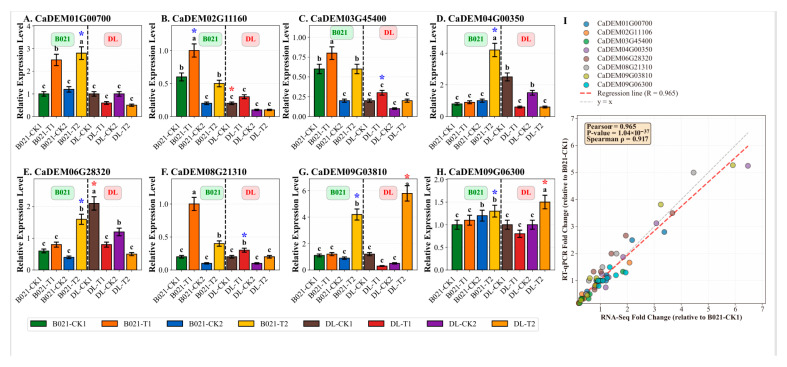
**Validation of key gene expression by RT-qPCR.** (**A**–**H**) Relative expression levels of eight selected genes determined by RT-qPCR across eight sample conditions. Data are presented as mean ± SD (n = 3). Different lowercase letters indicate significant differences (*p* < 0.05) within each genotype group (B021 or DL). Asterisks indicate significant differences compared to the respective control (B021-CK1 for B021 samples, DL-CK1 for DL samples): * *p* < 0.05. The vertical dashed line separates B021 (heat-tolerant) and DL (heat-sensitive) genotypes. (**A**) CaDEM01G00700 (HSP70), (**B**) CaDEM02G11160, (**C**) CaDEM03G45400, (**D**) CaDEM04G00350, (**E**) CaDEM06G28320, (**F**) CaDEM08G21310, (**G**) CaDEM09G03810, and (**H**) CaDEM09G06300. (**I**) Correlation analysis between RNA-Seq and RT-qPCR fold-change values. Each point represents a gene–sample combination (n = 64). The red dashed line indicates the regression line (R = 0.965, *p* < 0.001). The gray dashed line represents y = x (perfect agreement).

The RT-qPCR results for the eight selected genes are presented in Figure 6A–H, showing their relative expression levels across different sample conditions. The expression patterns revealed genotype- and treatment-specific responses. For instance, *CaDEM01G00700* (HSP70 family gene) showed significant upregulation in B021-T2 (Figure 6A), while CaDEM04G00350 exhibited dramatic induction in B021-T2 (Figure 6D). *CaDEM09G03810* displayed the highest expression in DL-T2 among all conditions (Figure 6G). These results showed a high degree of concordance with the RNA-seq expression profiles. To provide robust quantitative validation, we calculated fold-change values for both RNA-Seq and RT-qPCR data relative to the B021-CK1 control condition.

Statistical correlation analysis revealed a strong positive correlation between RNA-Seq and RT-qPCR fold-change values. Pearson correlation coefficient (r) was 0.965 (*p* < 0.001), and Spearman correlation coefficient (ρ) was 0.917 (*p* < 0.001) (Figure 6I). These very high correlation coefficients (r > 0.9) provide exceptionally strong statistical support for the reliability and accuracy of our high-throughput sequencing data. Specifically, the fold-change comparisons demonstrated consistent expression patterns between RNA-Seq and RT-qPCR results. For example, *CaDEM01G00700* (HSP70 family gene) showed a 3.38-fold increase in RNA-Seq and a 2.8-fold increase in RT-qPCR in the B021-T2 sample relative to B021-CK1. *CaDEM04G00350* exhibited a 6.47-fold increase in RNA-Seq and a 5.25-fold increase in RT-qPCR in B021-T2. *CaDEM09G03810* showed a 5.91-fold increase in RNA-Seq and a 5.27-fold increase in RT-qPCR in DL-T2. These quantitative comparisons, along with the very high correlation coefficients, confirm that genes significantly up-regulated by heat stress in B021 also exhibited increased expression in RT-qPCR, while genes down-regulated in DL were similarly validated. The expression patterns of specific target genes of miR156 (often associated with developmental timing, such as the *SPL* transcription factors) showed consistent fold-change values between RNA-Seq and RT-qPCR, with notable differences, especially between heat-tolerant and sensitive genotypes. Similarly, the target genes of miR166 (known to affect organogenesis, e.g., class III HD-Zip transcription factors) showed differential regulation patterns that were validated by both methods, suggesting that they play a role in development and morphogenesis under heat stress.

Functional categorization of these eight validated genes revealed that they are involved in key processes such as protein folding (HSP70/90), stress signaling (CAT, POD, and SOD), hormone biosynthesis, cell-wall modification, and primary metabolism. These categories are consistent with the results of the broader functional enrichment analysis in Section 3.7, further emphasizing their importance in the pepper heat stress response. For example, validated genes associated with heat shock proteins (CaDEM01G00700) show strong induction in B021 but diminished response in DL, whereas genes involved in specific hormonal pathways (e.g., jasmonic acid biosynthesis or signaling) are differentially regulated in a genotype- and developmental stage-specific manner. In addition, the expression patterns of several core genes in the WGCNA network (including genes directly linked to miR156/miR166 regulatory nodes) were also validated, which further highlighted their central role in the complex heat stress adaptation network (Figure 6A–H). This comprehensive validation with quantitative fold-change comparisons and statistical correlation analysis provides strong experimental support for the molecular changes identified by our multi-omics approach.

### 3.7. Enzymatic Quantification of Antioxidant Remodeling Under Heat

**Figure 7:**
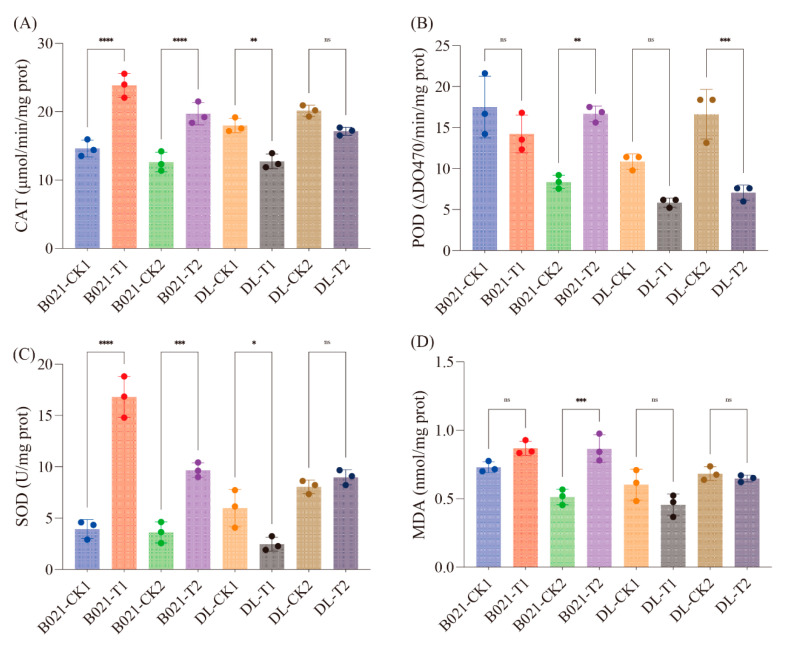
**Antioxidant enzyme activities and lipid peroxidation in pepper anthers under heat.** Activities of (**A**) catalase (CAT, μmol min^−1^ mg^−1^ protein), (**B**) peroxidase (POD, ΔOD_470_ min^−1^ mg^−1^ protein), and (**C**) superoxide dismutase (SOD, U mg^−1^ protein), and levels of (**D**) malondialdehyde (MDA, nmol mg^−1^ protein) across genotypes (B021, DL), stages (T1, T2), and treatments (T, HT). Symbols denote statistical significance: ns = not significant (p>0.05), * = significant at p≤0.05, ** = significant at p≤0.01, *** = significant at p≤0.001, **** = significant at p≤0.0001.

Guided by the transcriptome/miRNA evidence for ROS-related reprogramming, we quantified antioxidant capacity using commercial ELISA kits. Catalase (CAT) increased significantly in B021 at both stages and rose modestly in DL at T1 but not T2 (Figure 7A). Peroxidase (POD) displayed stage-dependent induction, being significantly higher at T2 in both genotypes but unchanged at T1 (Figure 7B). Superoxide dismutase (SOD) was robustly induced in B021 at both stages, while DL exhibited a smaller increase at T1 and no change at T2 (Figure 7C). Malondialdehyde (MDA), a lipid peroxidation proxy, showed a significant increase only in B021 at T2, whereas the other three contrasts were not significant (Figure 7D). Collectively, these measurements corroborate a genotype- and stage-specific reinforcement of enzymatic ROS scavenging under heat, with the tolerant background (B021) showing the most consistent induction across enzymes. Those results independently validate the multi-omics inference that antioxidant defenses are mobilized under heat stress and indicate that the tolerant genotype maintains a stronger and more sustained enzymatic response. The combination of CAT/SOD/POD induction with limited MDA accumulation across contrasts is consistent with more effective ROS homeostasis in B021, supporting its capacity to sustain anther function and dehiscence under elevated temperature.

## 4. Discussion

Our data establish that anther dehiscence in pepper is highly vulnerable to heat, but that tolerance is achievable when developmental, hormonal, and redox programs remain synchronized. The collapse of ADR in DL above 34–38 °C, contrasted with normal dehiscence in B021, is consistent with the recognized heat sensitivity of male reproduction and aligns with prior observations that developing anthers are among the most heat-labile tissues in flowering plants (Figure 1) [34]. The histological signatures we observed in DL—tapetal enlargement and premature degradation, locule contraction, and shriveled anthers—mirror classical heat-damage phenotypes reported across species, reinforcing the view that thermal injury derails the cellular architecture that normally enables anther opening (Figure 1).

A unifying interpretation emerges when the multi-omics layers are considered together. First, the tolerant genotype-maintained expression programs required for endothecial secondary-wall thickening and stomium competence, whereas the sensitive genotype down-weighted phenylpropanoid/secondary-wall and transport capacities early (PMC) and further suppressed wall biogenesis at tetrad (Figure S4). These trends dovetail with the genetic framework for dehiscence: secondary thickening of the endothecium is necessary to generate the tensile force that ruptures the stomium, a process controlled by MYB26 and downstream NACs (NST1/NST2). Perturbation of this axis leads to indehiscence in Arabidopsis and other systems, suggesting that the transcriptional trajectories we observed in DL are mechanistically sufficient to explain failed opening [35,36,37].

The small-RNA layer provides a plausible post-transcriptional mechanism for maintaining (or losing) this wall program under heat. We detected genotype- and stage-specific remodeling of conserved families with direct relevance to dehiscence (Figure 3 and Figure S5). Notably, the miR397-laccase module—well known to tune lignin deposition by targeting *LAC* genes—showed stronger degradome support in B021, consistent with more precise control of lignification timing rather than wholesale activation, which would otherwise risk ectopic stiffening (Figure 3B, Supplemental Tables S3–S5). Similar miR397–*LAC* regulation of lignin has been demonstrated across taxa, reinforcing the transferability of this axis as a breeding lever [38,39,40].

Hormonal integration appears to be a decisive layer. Our results implicate auxin nodes (miR167/miR160–ARF/TAS3) together with jasmonate-related modules (miR319/miR159–TCP/MYB), matching the established model in which ARF6/ARF8 promote JA production to drive filament elongation and anther dehiscence (Figure 3B and Figure 4). Heat is known to depress auxin homeostasis in developing anthers; thus, preserving auxin–JA crosstalk likely distinguishes B021 from DL [41,42]. The enrichment of diterpenoid/terpenoid and gibberellin terms in our target sets further supports a hormone-centric timing mechanism that coordinates wall maturation with pollen release [43,44].

Cross-cutting layers are redox and energy balance. Transcriptome and sRNA evidence pointed to respiratory and ROS-scavenging pathways, and ELISA-based assays confirmed enzyme-level reinforcement (SOD, CAT, and POD) with restrained lipid peroxidation in B021 (Figures S4 and Figure 7). This pattern is consistent with the broader literature, in which antioxidant systems buffer thermal ROS surges and preserve cellular function, thereby protecting developmental decisions such as dehiscence timing. The convergence we observed—TCA/respiration signals, ROS homeostasis, and proteostasis/ER–peroxisome quality control—suggests that tolerant anthers prioritize ATP supply and oxidative buffering to meet the mechanical and metabolic cost of wall remodeling under heat [45,46].

Taken together, we propose a model in which successful dehiscence under heat requires the coordinated operation of four modules: (i) endothecial secondary-wall assembly and stomium remodeling (maintained in B021, attenuated in DL); (ii) auxin–JA (and GA) crosstalk that gates the timing of dehiscence; (iii) respiratory/redox support that sustains energy and prevents ROS-driven damage; and (iv) protein/membrane quality control that stabilizes secretory and peroxisomal functions during stress. This organization is consistent with genetic studies placing MYB26/NST factors and hormone circuits at the core of dehiscence control, now extended here with post-transcriptional regulation by conserved miRNAs and enzyme-level validation of redox buffering. The miRNA–target axes identified here (e.g., miR397-LAC, miR167/160-ARF/TAS3, and miR319/159-TCP/MYB) provide tractable entry points for breeding and engineering heat-resilient pepper. Given the pleiotropy of these regulators, allele-specific tuning (e.g., editing miRNA binding sites in selected LAC/ARF/TCP paralogs) or stage-restricted promoters may minimize trade-offs. Two limitations should be addressed in future work: (i) cell-type resolution—because endothecium, tapetum, and stomium have distinct tasks, single-cell/spatial profiling would sharpen causal inference; and (ii) functional tests—CRISPR or target-mimicry validation of priority axes in pepper backgrounds is needed to confirm sufficiency for dehiscence rescue under heat. Nevertheless, by integrating histology, multi-omics, and biochemical assays, our study delineates a coherent, heat-responsive regulatory architecture for anther opening that is strongly supported by prior genetic and physiological frameworks.

Components of our four-module coordination framework align with mechanisms previously characterized by Arabidopsis and other model systems. For example, endothecial secondary-wall formation/cell-wall remodeling is a well-established prerequisite for stomium rupture and anther opening, and jasmonate-centered hormonal control (with interactions involving auxin and gibberellin) has been extensively implicated in late stamen maturation and dehiscence. Likewise, ROS/redox homeostasis has been linked to anther development and dehiscence-associated wall modification, and protein/membrane quality control is a recognized requirement for reproductive thermotolerance under heat stress. Importantly, our study provides pepper-specific, integrative support that these processes operate as a coordinated, genotype- and stage-resolved program during heat challenge. First, across two developmental stages and two contrasting genotypes, independent data layers converge on the same four modules—energy/redox, hormone/terpenoid signaling, vascular–cell-wall programs, and membrane/proteostasis–osmotic buffering—rather than implicating these pathways in isolation. Second, the inclusion of degradome evidence supplies in vivo support for miRNA-guided cleavage that links regulatory small RNAs to module components in pepper, strengthening causal connections beyond transcript abundance alone. Third, WGCNA identifies trait-associated modules and hub-gene sets that map onto the same functional axes, while physiological enzyme/oxidative readouts further corroborate the predicted redox-buffering behavior under heat. Together, these integrative results extend model-plant knowledge by demonstrating a coordinated four-module architecture in Capsicum annuum and by nominating concrete pepper candidates for breeding heat-resilient anther dehiscence.

## 5. Conclusions

In this study, we dissected why pepper anthers failed or succeeded in opening under heat. The sensitive cultivar (DL) exhibited catastrophic loss of dehiscence, whereas the tolerant landrace (B021) maintained normal anther opening. Transcriptome profiling revealed early suppression of phenylpropanoid/cell-wall and proteostasis pathways in DL, while B021 sustained reproductive and stress-integration programs. Small-RNA and degradome analyses identified conserved, degradome-supported miRNA–mRNA axes (including miR397–*laccase*, miR167/miR160–ARF/TAS3, and miR319/miR159–TCP/MYB) that aligned with lignification, wall remodeling, and hormone crosstalk. Co-expression modules and hub genes converged on these processes, and enzyme assays corroborated stronger antioxidant capacity in B021. Together, these findings define a coordinated regulatory architecture that supports anther dehiscence under heat and explain the genotype contrast. They provide tractable molecular targets—both genes and their controlling miRNAs—for breeding heat-resilient pepper.

## Data Availability

Data are available on request.

## Supplementary Materials

The following supporting information can be downloaded at https://www.mdpi.com/article/10.3390/biology15020129/s1: Table S1: Summary of transcriptome sequencing data generated for 24 samples using Illumina sequencing platform. Table S2: Complete list of differentially expressed genes (DEGs) regulated by high temperature in pepper flower buds. Table S3: Complete list of differentially expressed miRNAs (DEmiRs) regulated by high temperature in pepper flower buds. Table S4: The results of the targets identified with degradome analysis in B021. Table S5: The results of the targets identified with degradome analysis in DL. Table S6: primers used in this study. Figure S1: Morphological characteristics of pepper anthers under control and heat stress conditions. Figure S2: Sample-to-sample correlation analysis of RNA-seq data. A heatmap showing pairwise Pearson correlation coefficients (r) among all RNA-seq samples from the two pepper cultivars (DL and B021) across different treatments/stages. Figure S3: Differential expression analysis and sample clustering of RNA-seq data. (A–D) Volcano plots showing differentially expressed genes (DEGs) in B021_T1_vs_CK1, B021_T2_vs_CK2, DL_T1_vs_CK1, and DL_T2_vs_CK2 comparisons, respectively. Red and blue dots indicate up-regulated and down-regulated genes, respectively. (E–H) Heatmaps and hierarchical clustering of DEGs for the corresponding comparisons, showing clear expression patterns and consistency among biological replicates. Figure S4: Differential gene expression and functional enrichment analysis under high-temperature stress. (A) GO enrichment results for different comparisons, and (B) KEGG enrichment results for different comparisons. Figure S5: Heat-responsive miRNA remodeling and degradome-supported targeting in pepper anthers. Unsupervised clustering heatmap of DEmiRs across all 24 libraries (columns, samples, rows, miRNAs, color scale, and z-scored abundance), illustrating genotype- and stage-dependent patterns among conserved families implicated in anther biology. Figure S6: Functional organization of DEmiR targets into process-level modules under heat. (A,B) Enrichment analysis of targets of differentially expressed miRNAs (DEmiRs) across the four contrasts, highlighting over-represented processes linked to (A) GO enrichment analysis and (B) KEGG enrichment analysis.
