# Supplementary material for: An Integrative Small RNA–Degradome–Transcriptome Analysis Reveals Mechanisms of Heat-Induced Anther Indehiscence in Pepper
Source: Biology (Basel). 2026 Jan 12;15(2):129. doi: 10.3390/biology15020129 (PMC12838170; doi:10.3390/biology15020129)
Supplement: Supplementary file 1 [file biology-15-00129-s001.zip › Figure S2.pdf]

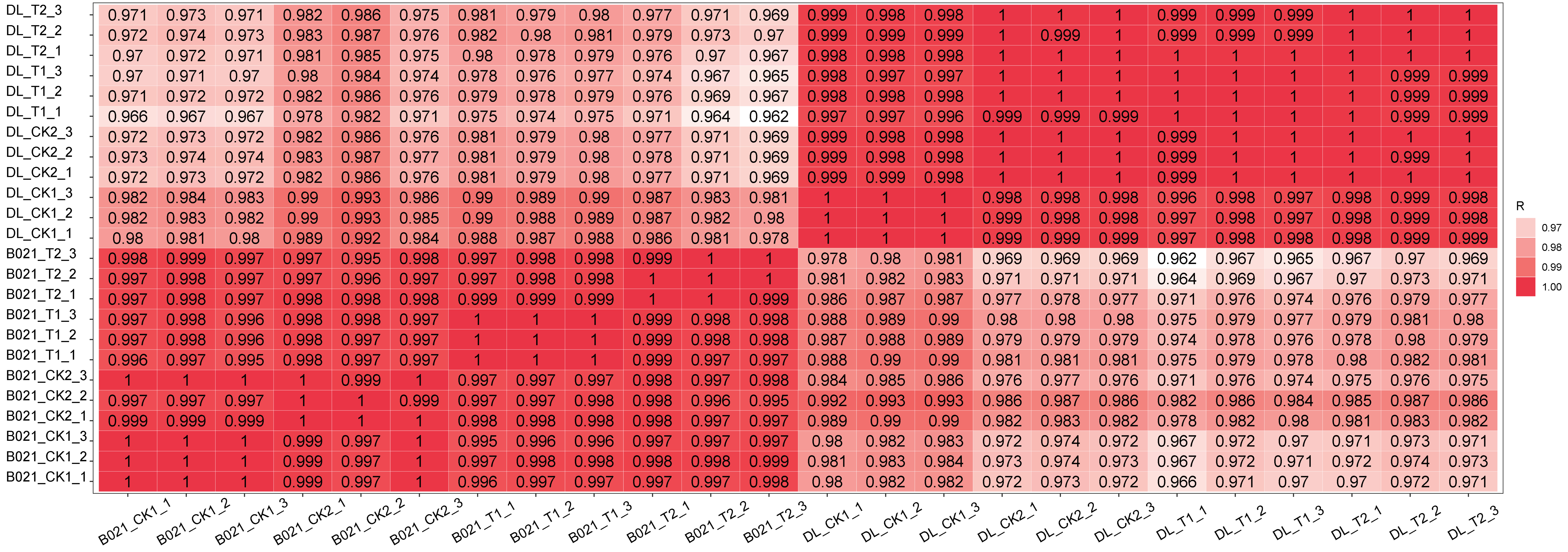
DL_T2_3 0.971 0.973 0.971 0.982 0.986 0.975 0.981 0.979 0.98 0.977 0.971 0.969 0.999 0.998 0.998 1 1 1 0.999 0.999 0.999 1 1 1
DL_T2_2 0.972 0.974 0.973 0.983 0.987 0.976 0.982 0.98 0.981 0.979 0.973 0.97 0.999 0.999 0.999 1 0.999 1 0.999 0.999 0.999 1 1 1
DL_T2_1 0.97 0.972 0.971 0.981 0.985 0.975 0.98 0.978 0.979 0.976 0.97 0.967 0.998 0.998 0.998 1 1 1 1 1 1 1 1 1
DL_T1_3 0.97 0.971 0.97 0.98 0.984 0.974 0.978 0.976 0.977 0.974 0.967 0.965 0.998 0.997 0.997 1 1 1 1 1 1 1 0.999 0.999
DL_T1_2 0.971 0.972 0.972 0.982 0.986 0.976 0.979 0.978 0.979 0.976 0.969 0.967 0.998 0.998 0.998 1 1 1 1 1 1 1 0.999 0.999
DL_T1_1 0.966 0.967 0.967 0.978 0.982 0.971 0.975 0.974 0.975 0.971 0.964 0.962 0.997 0.997 0.996 0.999 0.999 0.999 1 1 1 1 0.999 0.999
DL_CK2_3 0.972 0.973 0.972 0.982 0.986 0.976 0.981 0.979 0.98 0.977 0.971 0.969 0.999 0.998 0.998 1 1 1 0.999 1 1 1 1 1
DL_CK2_2 0.973 0.974 0.974 0.983 0.987 0.977 0.981 0.979 0.98 0.978 0.971 0.969 0.999 0.998 0.998 1 1 1 0.999 1 1 1 0.999 1
DL_CK2_1 0.972 0.973 0.972 0.982 0.986 0.976 0.981 0.979 0.98 0.977 0.971 0.969 0.999 0.999 0.998 1 1 1 0.999 1 1 1 1 1
DL_CK1_3 0.982 0.984 0.983 0.99 0.993 0.986 0.99 0.989 0.99 0.987 0.983 0.981 1 1 1 0.998 0.998 0.998 0.996 0.998 0.997 0.998 0.999 0.998
DL_CK1_2 0.982 0.983 0.982 0.99 0.993 0.985 0.99 0.988 0.989 0.987 0.982 0.98 1 1 1 0.999 0.998 0.998 0.997 0.998 0.997 0.998 0.999 0.998
DL_CK1_1 0.98 0.981 0.98 0.989 0.992 0.984 0.988 0.987 0.988 0.986 0.981 0.978 1 1 1 0.999 0.999 0.999 0.997 0.998 0.998 0.998 0.999 0.999
B021_T2_3 0.998 0.999 0.997 0.997 0.995 0.998 0.997 0.998 0.998 0.999 1 1 0.978 0.98 0.981 0.969 0.969 0.969 0.962 0.967 0.965 0.967 0.97 0.969
B021_T2_2 0.997 0.998 0.997 0.997 0.996 0.997 0.997 0.998 0.998 1 1 1 0.981 0.982 0.983 0.971 0.971 0.971 0.964 0.969 0.967 0.97 0.973 0.971
B021_T2_1 0.997 0.998 0.997 0.998 0.998 0.998 0.999 0.999 0.999 1 1 0.999 0.986 0.987 0.987 0.977 0.978 0.977 0.971 0.976 0.974 0.976 0.979 0.977
B021_T1_3 0.997 0.998 0.996 0.998 0.998 0.997 1 1 1 0.999 0.998 0.998 0.988 0.989 0.99 0.98 0.98 0.98 0.975 0.979 0.977 0.979 0.981 0.98
B021_T1_2 0.997 0.998 0.996 0.998 0.997 0.997 1 1 1 0.999 0.998 0.998 0.987 0.988 0.989 0.979 0.979 0.979 0.974 0.978 0.976 0.978 0.98 0.979
B021_T1_1 0.996 0.997 0.995 0.998 0.997 0.997 1 1 1 0.999 0.997 0.997 0.988 0.99 0.99 0.981 0.981 0.981 0.975 0.979 0.978 0.98 0.982 0.981
B021_CK2_3 1 1 1 1 0.999 1 0.997 0.997 0.997 0.998 0.997 0.998 0.984 0.985 0.986 0.976 0.977 0.976 0.971 0.976 0.974 0.975 0.976 0.975
B021_CK2_2 0.997 0.997 0.997 1 1 0.999 0.997 0.997 0.998 0.998 0.996 0.995 0.992 0.993 0.993 0.986 0.987 0.986 0.982 0.986 0.984 0.985 0.987 0.986
B021_CK2_1 0.999 0.999 0.999 1 1 1 0.998 0.998 0.998 0.998 0.997 0.997 0.989 0.99 0.99 0.982 0.983 0.982 0.978 0.982 0.98 0.981 0.983 0.982
B021_CK1_3 1 1 1 0.999 0.997 1 0.995 0.996 0.996 0.997 0.997 0.997 0.98 0.982 0.983 0.972 0.974 0.972 0.967 0.972 0.97 0.971 0.973 0.971
B021_CK1_2 1 1 1 0.999 0.997 1 0.997 0.998 0.998 0.998 0.998 0.999 0.981 0.983 0.984 0.973 0.974 0.973 0.967 0.972 0.971 0.972 0.974 0.973
B021_CK1_1 1 1 1 0.999 0.997 1 0.996 0.997 0.997 0.997 0.997 0.998 0.98 0.982 0.982 0.972 0.973 0.972 0.966 0.971 0.97 0.97 0.972 0.971
B021_CK1_1 B021_CK1_2 B021_CK1_3 B021_CK2_1 B021_CK2_2 B021_CK2_3 B021_T1_1 B021_T1_2 B021_T1_3 B021_T2_1 B021_T2_2 B021_T2_3 DL_CK1_1 DL_CK1_2 DL_CK1_3 DL_CK2_1 DL_CK2_2 DL_CK2_3 DL_T1_1 DL_T1_2 DL_T1_3 DL_T2_1 DL_T2_2 DL_T2_3
R
0.97
0.98
0.99
1.00

	B021_CK1_1	B021_CK1_2	B021_CK1_3	B021_CK2_1	B021_CK2_2	B021_CK2_3	B021_T1_1	B021_T1_2	B021_T1_3	B021_T2_1	B021_T2_2	B021_T2_3	DL_CK1_1	DL_CK1_2	DL_CK1_3	DL_CK2_1	DL_CK2_2	DL_CK2_3	DL_T1_1	DL_T1_2	DL_T1_3	DL_T2_1	DL_T2_2	DL_T2_3
DL_T2_3	0.971	0.973	0.971	0.982	0.986	0.975	0.981	0.979	0.98	0.977	0.971	0.969	0.999	0.998	0.998	1	1	1	0.999	0.999	0.999	1	1	1
DL_T2_2	0.972	0.974	0.973	0.983	0.987	0.976	0.982	0.98	0.981	0.979	0.973	0.97	0.999	0.999	0.999	1	0.999	1	0.999	0.999	0.999	1	1	1
DL_T2_1	0.97	0.972	0.971	0.981	0.985	0.975	0.98	0.978	0.979	0.976	0.97	0.967	0.998	0.998	0.998	1	1	1	1	1	1	1	1	1
DL_T1_3	0.97	0.971	0.97	0.98	0.984	0.974	0.978	0.976	0.977	0.974	0.967	0.965	0.998	0.997	0.997	1	1	1	1	1	1	1	0.999	0.999
DL_T1_2	0.971	0.972	0.972	0.982	0.986	0.976	0.979	0.978	0.979	0.976	0.969	0.967	0.998	0.998	0.998	1	1	1	1	1	1	1	0.999	0.999
DL_T1_1	0.966	0.967	0.967	0.978	0.982	0.971	0.975	0.974	0.975	0.971	0.964	0.962	0.997	0.997	0.996	0.999	0.999	0.999	1	1	1	1	0.999	0.999
DL_CK2_3	0.972	0.973	0.972	0.982	0.986	0.976	0.981	0.979	0.98	0.977	0.971	0.969	0.999	0.998	0.998	1	1	1	0.999	1	1	1	1	1
DL_CK2_2	0.973	0.974	0.974	0.983	0.987	0.977	0.981	0.979	0.98	0.978	0.971	0.969	0.999	0.998	0.998	1	1	1	0.999	1	1	1	0.999	1
DL_CK2_1	0.972	0.973	0.972	0.982	0.986	0.976	0.981	0.979	0.98	0.977	0.971	0.969	0.999	0.999	0.998	1	1	1	0.999	1	1	1	1	1
DL_CK1_3	0.982	0.984	0.983	0.99	0.993	0.986	0.99	0.989	0.99	0.987	0.983	0.981	1	1	1	0.998	0.998	0.998	0.996	0.998	0.997	0.998	0.999	0.998
DL_CK1_2	0.982	0.983	0.982	0.99	0.993	0.985	0.99	0.988	0.989	0.987	0.982	0.98	1	1	1	0.999	0.998	0.998	0.997	0.998	0.997	0.998	0.999	0.998
DL_CK1_1	0.98	0.981	0.98	0.989	0.992	0.984	0.988	0.987	0.988	0.986	0.981	0.978	1	1	1	0.999	0.999	0.999	0.997	0.998	0.998	0.998	0.999	0.999
B021_T2_3	0.998	0.999	0.997	0.997	0.995	0.998	0.997	0.998	0.998	0.999	1	1	0.978	0.98	0.981	0.969	0.969	0.969	0.962	0.967	0.965	0.967	0.97	0.969
B021_T2_2	0.997	0.998	0.997	0.997	0.996	0.997	0.997	0.998	0.998	1	1	1	0.981	0.982	0.983	0.971	0.971	0.971	0.964	0.969	0.967	0.97	0.973	0.971
B021_T2_1	0.997	0.998	0.997	0.998	0.998	0.998	0.999	0.999	0.999	1	1	0.999	0.986	0.987	0.987	0.977	0.978	0.977	0.971	0.976	0.974	0.976	0.979	0.977
B021_T1_3	0.997	0.998	0.996	0.998	0.998	0.997	1	1	1	0.999	0.998	0.998	0.988	0.989	0.99	0.98	0.98	0.98	0.975	0.979	0.977	0.979	0.981	0.98
B021_T1_2	0.997	0.998	0.996	0.998	0.997	0.997	1	1	1	0.999	0.998	0.998	0.987	0.988	0.989	0.979	0.979	0.979	0.974	0.978	0.976	0.978	0.98	0.979
B021_T1_1	0.996	0.997	0.995	0.998	0.997	0.997	1	1	1	0.999	0.997	0.997	0.988	0.99	0.99	0.981	0.981	0.981	0.975	0.979	0.978	0.98	0.982	0.981
B021_CK2_3	1	1	1	1	0.999	1	0.997	0.997	0.997	0.998	0.997	0.998	0.984	0.985	0.986	0.976	0.977	0.976	0.971	0.976	0.974	0.975	0.976	0.975
B021_CK2_2	0.997	0.997	0.997	1	1	0.999	0.997	0.997	0.998	0.998	0.996	0.995	0.992	0.993	0.993	0.986	0.987	0.986	0.982	0.986	0.984	0.985	0.987	0.986
B021_CK2_1	0.999	0.999	0.999	1	1	1	0.998	0.998	0.998	0.998	0.997	0.997	0.989	0.99	0.99	0.982	0.983	0.982	0.978	0.982	0.98	0.981	0.983	0.982
B021_CK1_3	1	1	1	0.999	0.997	1	0.995	0.996	0.996	0.997	0.997	0.997	0.98	0.982	0.983	0.972	0.974	0.972	0.967	0.972	0.97	0.971	0.973	0.971
B021_CK1_2	1	1	1	0.999	0.997	1	0.997	0.998	0.998	0.998	0.998	0.999	0.981	0.983	0.984	0.973	0.974	0.973	0.967	0.972	0.971	0.972	0.974	0.973
B021_CK1_1	1	1	1	0.999	0.997	1	0.996	0.997	0.997	0.997	0.997	0.998	0.98	0.982	0.982	0.972	0.973	0.972	0.966	0.971	0.97	0.97	0.972	0.971

R: 0.97, 0.98, 0.99, 1.00

Figure S2. Sample-to-sample correlation analysis of RNA-seq data. A heatmap showing pairwise Pearson correlation coefficients (r) among all RNA-seq samples from the two pepper cultivars (DL and B021) across different treatments/stages (e.g., CK and T).
