# Supplementary material for: An Integrative Small RNA–Degradome–Transcriptome Analysis Reveals Mechanisms of Heat-Induced Anther Indehiscence in Pepper
Source: Biology (Basel). 2026 Jan 12;15(2):129. doi: 10.3390/biology15020129 (PMC12838170; doi:10.3390/biology15020129)
Supplement: Supplementary file 1 [file biology-15-00129-s001.zip › Figure S3.pdf]

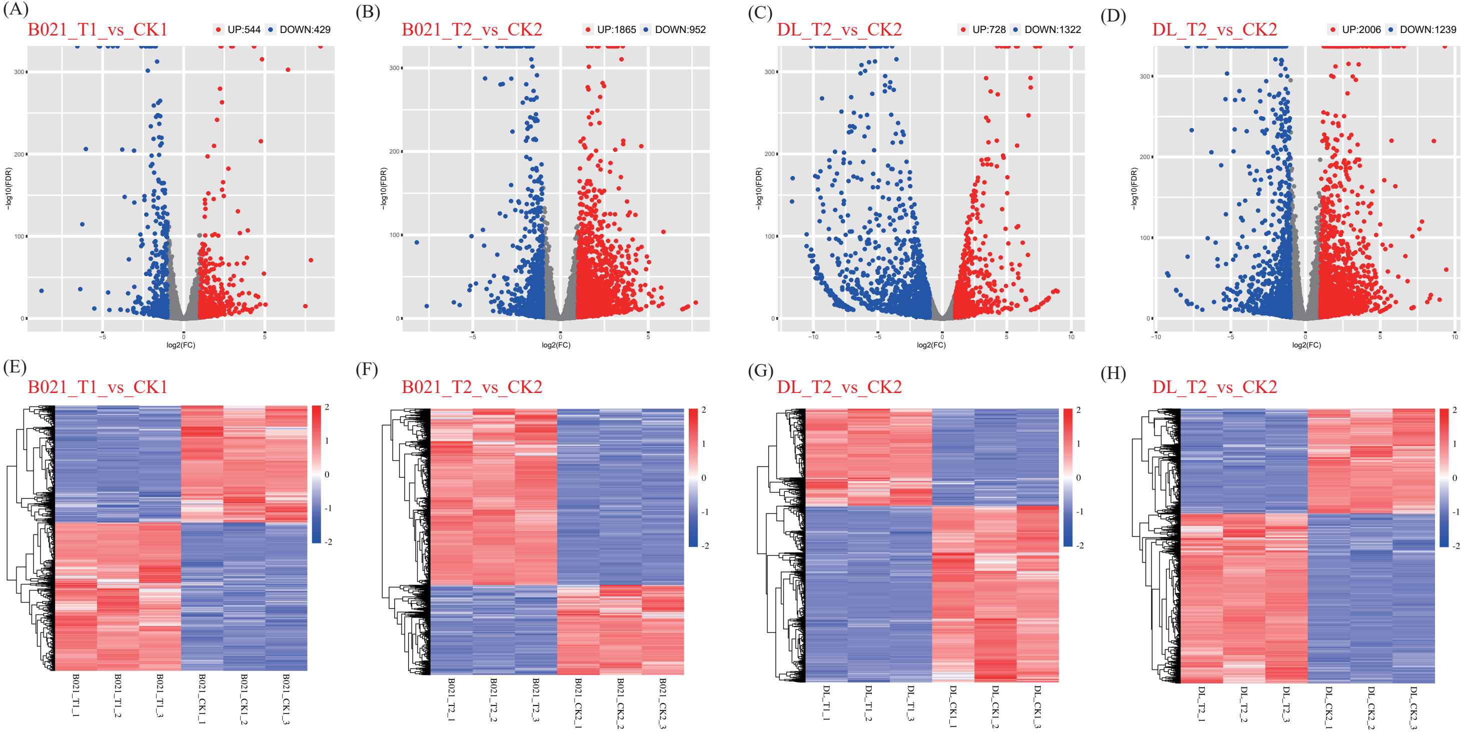

Figure S3. Differential expression analysis and sample clustering of RNA-seq data. (A–D) Volcano plots showing differentially expressed genes (DEGs) in B021_T1_vs_CK1, B021_T2_vs_CK2, DL_T1_vs_CK1, and DL_T2_vs_CK2 comparisons, respectively. Red and blue dots indicate up-regulated and down-regulated genes, respectively. (E–H) Heatmaps and hierarchical clustering of DEGs for the corresponding comparisons, showing clear expression patterns and consistency among biological replicates.
