# Supplementary material for: An Integrative Small RNA–Degradome–Transcriptome Analysis Reveals Mechanisms of Heat-Induced Anther Indehiscence in Pepper
Source: Biology (Basel). 2026 Jan 12;15(2):129. doi: 10.3390/biology15020129 (PMC12838170; doi:10.3390/biology15020129)
Supplement: Supplementary file 1 [file biology-15-00129-s001.zip › Figure S5.pdf]

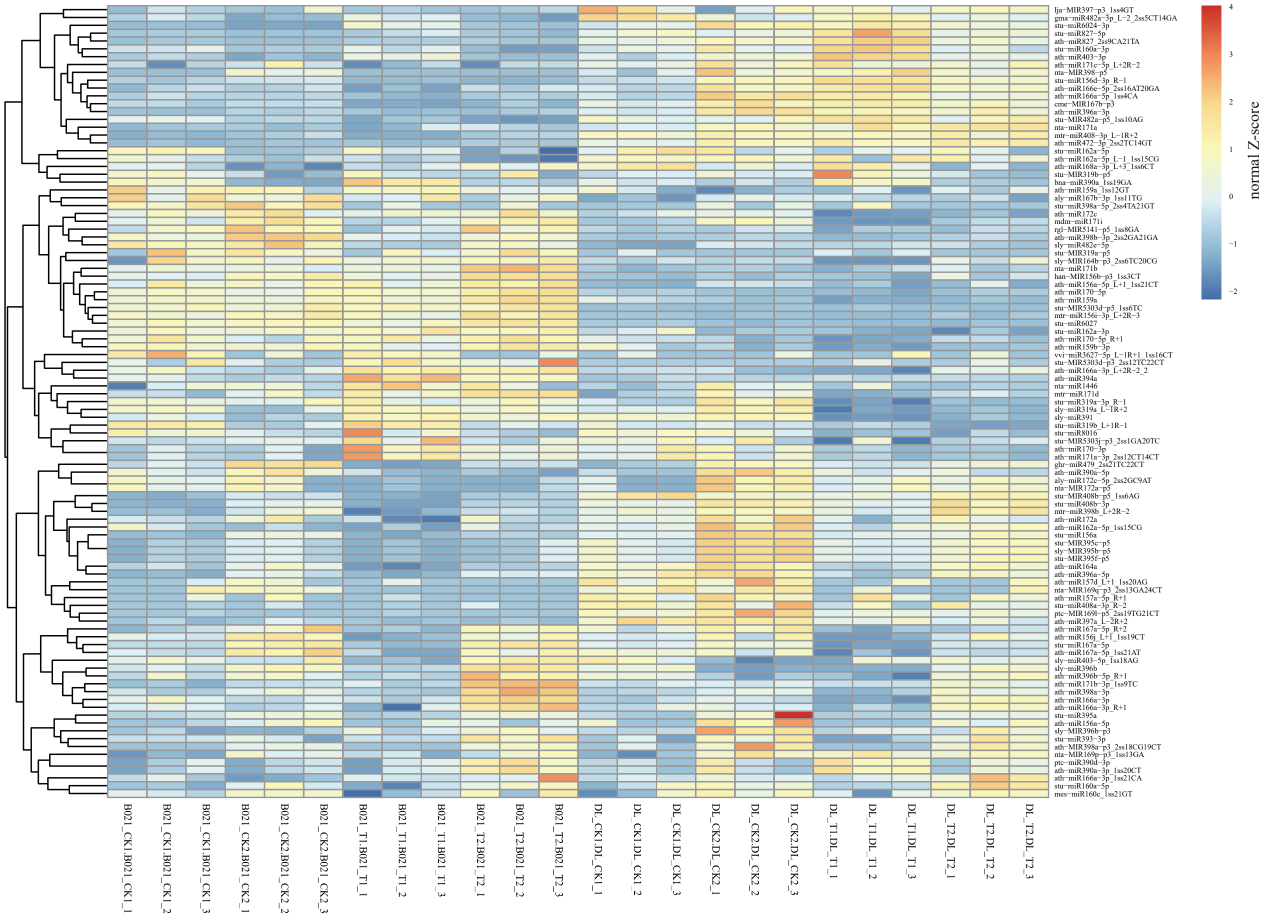

Figure S5. Heat-responsive miRNA remodeling and degradome-supported targeting in pepper anthers. Unsupervised clustering heatmap of DEmiRs across all 24 libraries (columns, samples, rows, miRNAs, color scale, z-scored abundance), illustrating genotype- and stage-dependent patterns among conserved families implicated in anther biology.
