# Supplementary figures and images for: An Integrative Small RNA–Degradome–Transcriptome Analysis Reveals Mechanisms of Heat-Induced Anther Indehiscence in Pepper
Source: Biology (Basel). 2026 Jan 12;15(2):129. doi: 10.3390/biology15020129 (PMC12838170; doi:10.3390/biology15020129)

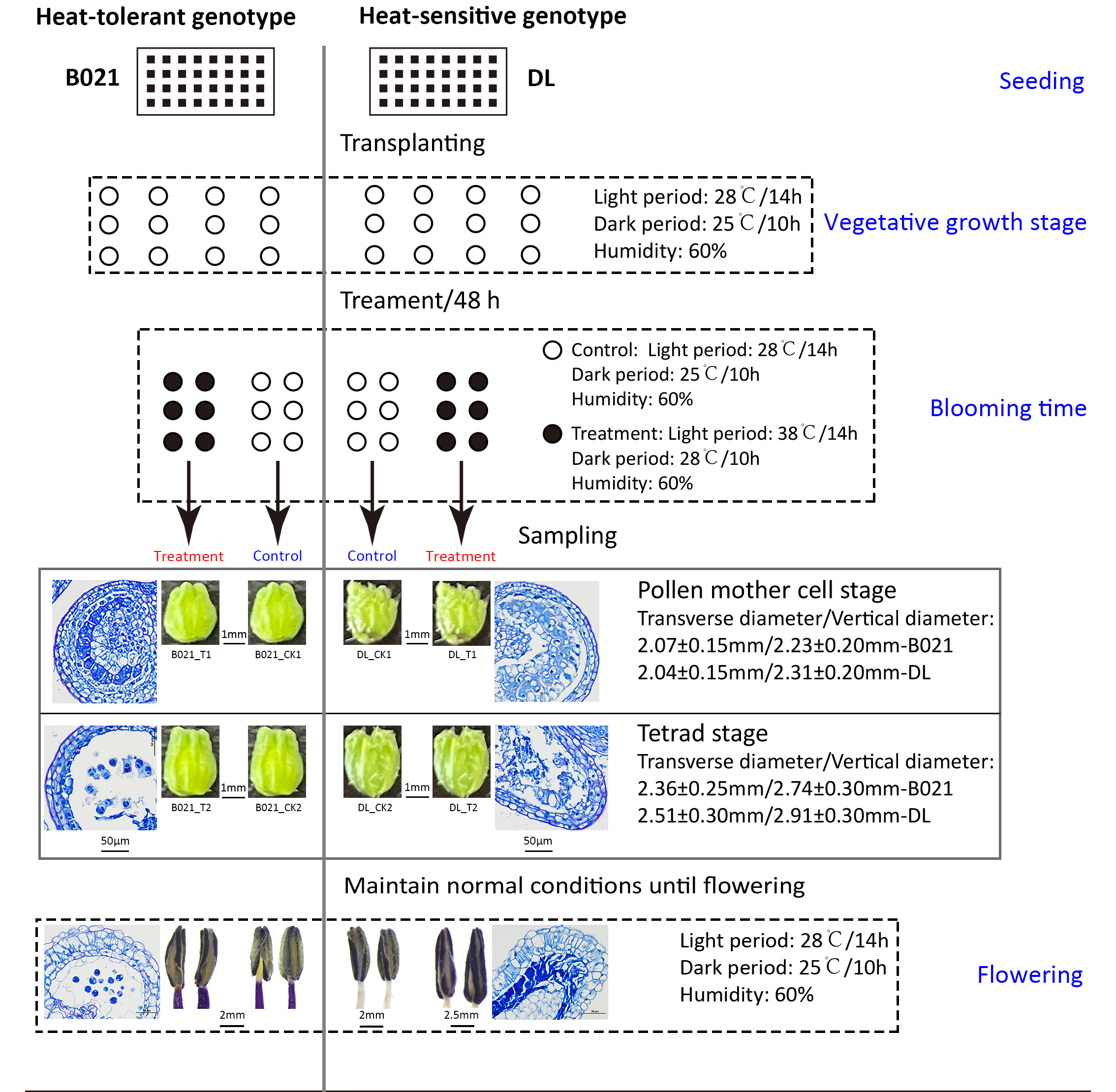

Supplement: Supplementary file 1 [file biology-15-00129-s001.zip › Figure S1.tif]
