# Supplementary material for: An Integrative Small RNA–Degradome–Transcriptome Analysis Reveals Mechanisms of Heat-Induced Anther Indehiscence in Pepper
Source: Biology (Basel). 2026 Jan 12;15(2):129. doi: 10.3390/biology15020129 (PMC12838170; doi:10.3390/biology15020129)
Supplement: Supplementary file 1 [file Figure S4.pdf › Figure S4.pdf]

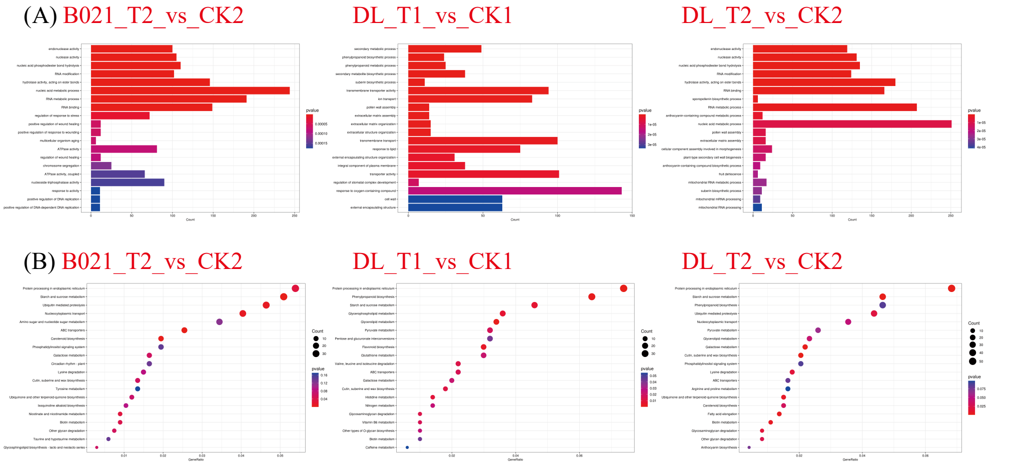

Figure S4. Differential gene expression and functional enrichment analysis under high temperature stress. (A) GO enrichment results for different comparisons, (B) KEGG enrichment results for different comparisons.
