# Supplementary material for: An Integrative Small RNA–Degradome–Transcriptome Analysis Reveals Mechanisms of Heat-Induced Anther Indehiscence in Pepper
Source: Biology (Basel). 2026 Jan 12;15(2):129. doi: 10.3390/biology15020129 (PMC12838170; doi:10.3390/biology15020129)
Supplement: Supplementary file 1 [file Figure S6.pdf › Figure S6.pdf]

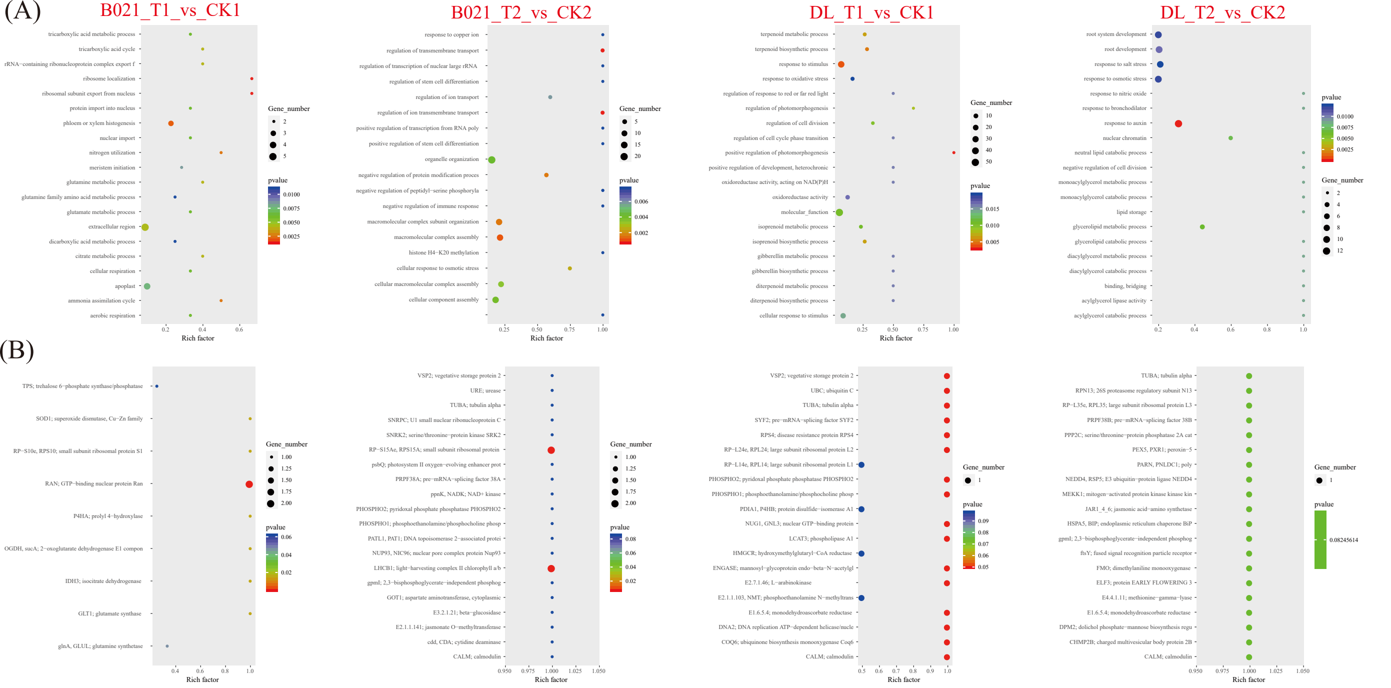

Figure S6. Functional enrichment analysis of DEmiR targets into process-level modules under heat. Enrichment analysis of targets of differentially expressed miRNAs (DEmiRs) across the four contrasts, highlighting over-represented processes linked to (A) GO enrichment analysis, (B) KEGG enrichment analysis.
